# The Expression Pattern of Genes Related to Melanogenesis and Endogenous Opioids in Psoriasis

**DOI:** 10.3390/ijms222313056

**Published:** 2021-12-02

**Authors:** Ulvi Loite, Liisi Raam, Ene Reimann, Paula Reemann, Ele Prans, Tanel Traks, Eero Vasar, Helgi Silm, Külli Kingo, Sulev Kõks

**Affiliations:** 1 Department of Dermatology and Venerology, University of Tartu, 31 Raja, 50417 Tartu, Estonia; ulvi.loite@ut.ee (U.L.); liisi.raam@kliinikum.ee (L.R.); paula.reemann@kliinikum.ee (P.R.); helgi.silm@kliinikum.ee (H.S.); kylli.kingo@kliinikum.ee (K.K.); 2 Dermatology Clinic, Tartu University Hospital, 31 Raja, 50417 Tartu, Estonia; 3 Institute of Genomics, University of Tartu, 23b/2 Riia, 51010 Tartu, Estonia; ene.reimann@ut.ee; 4 Department of Anaesthesiology and Intensive Care, Tartu University Hospital, 8 L. Puusepa, 51014 Tartu, Estonia; ele.prans@kliinikum.ee; 5 Department of Physiology, University of Tartu, 19 Ravila Street, 50411 Tartu, Estonia; eero.vasar@ut.ee; 6 The Perron Institute for Neurological and Translational Science, 8 Verdun St., Nedlands, WA 6009, Australia; sulev.koks@perron.uwa.edu.au; 7 Centre for Comparative Genomics, Murdoch University, 90 South St., Murdoch, WA 6150, Australia

**Keywords:** psoriasis, melanocortin system, melanogenesis enzymes, endogenous opioid system, mRNA expression analysis

## Abstract

The melanocortin system is a major regulator of stress responses in the skin and is responsible for the induction of melanin synthesis through activation of melanogenesis enzymes. The expression of both melanocortin system genes and melanogenesis enzyme genes is altered in psoriasis, and the focus here was on twelve genes related to the signal transduction between them. Additionally, five endogenous opioid system genes that are involved in cutaneous inflammation were examined. Quantitative real-time-PCR was utilized to measure mRNA expression in punch biopsies from lesional and non-lesional skin of psoriasis patients and from the skin of healthy control subjects. Most of the genes related to melanogenesis were down-regulated in patients (CREB1, MITF, LEF1, USF1, MAPK14, ICAM1, PIK3CB, RPS6KB1, KIT, and ATRN). Conversely, an up-regulation occurred in the case of opioids (PENK, PDYN, and PNOC). The suppression of genes related to melanogenesis is in agreement with the reported reduction in pigmentation signaling in psoriatic skin and potentially results from the pro-inflammatory environment. The increase in endogenous opioids can be associated with their involvement in inflammatory dysregulation in psoriasis.

## 1. Introduction

Psoriasis is a chronic, relapsing, inflammatory skin disease that generally affects around 2–4% of the population in Western countries [1,2]. Patients display inflamed erythematous plaques covered with silvery-white dry scales that are located mainly on elbows, knees, scalp, and in the lumbosacral area. The main histological characteristics include the abnormal proliferation of keratinocytes and infiltration of immune cells, predominantly T cells and dendritic cells, in psoriatic lesions [3]. An imbalance of cytokines has been described in psoriasis, with the tumor necrosis factor (TNF)-α and interleukin (IL)-23/T-helper (Th)17 pathway bearing a central role in pathogenesis [4]. The estimated heritability is above 60%, which is high for a complex disease and valuable insights have been gained from genetic susceptibility loci of which there are currently over 60 for populations of European ancestry [5].

Skin is armed with a sophisticated local neuro-endocrine-immune system that encompasses classical neuropeptides and neuroendocrine regulators including a local melanocortin and opioid system, steroidogenic and secosteroidogenic, neuroimmune and metabolic activities [6,7]. Extensive crosstalk between epithelial, stromal and immune cells through different signal transduction pathways of the skin regulates local immune responses to maintain and restore homeostasis, and to prevent chronic disease [8]. The melanocortin system has been identified among these numerous intertwined networks, with proopiomelanocortin (POMC) as its central molecule. It is a highly hierarchical network composed of α-, β-, and γ-melanocyte-stimulating hormone (MSH) and adrenocorticotropin (ACTH), all four of which are derived from POMC, five melanocortin receptors (MCR1-5), and two melanocortin receptor antagonists: agouti signaling protein (ASIP) and agouti-related protein (AGRP). Since corticotropin-releasing hormone (CRH) activates POMC, it can also be expanded to the CRH-POMC system and viewed as a cutaneous equivalent of the hypothalamo–pituitary–adrenal (HPA) axis [9]. The function of the melanocortin system is to coordinate local responses to stress and in addition to pigmentation, it is also involved in immune regulation [10]. Anti-inflammatory and immunomodulatory effects through a variety of mechanisms have been shown with ACTH and α-MSH and to a lesser extent with β-MSH and γ-MSH, forming the basis for suggestions of therapeutic usage [10,11]. At the same time, inflammatory factors contribute to the regulation of melanogenesis. Interestingly, pro-inflammatory cytokines associated with psoriasis such as TNF, IL-17 and IL-6 suppress melanin synthesis, but others can also activate the process [12]. There appears to be an accompanying increase in melanocytes in psoriatic lesions, which explains the reversal of hypopigmentation that is seen after psoriasis treatment and even the subsequent development of hyperpigmentation [13,14,15]. In our previous study, we showed significant alterations in the expression of genes of the melanocortin system in the skin of psoriasis patients [16]. The decreased levels of two genes involved in melanin synthesis, tyrosinase (TYR) and tyrosinase-related protein-1 (TYRP1) were also demonstrated in that report. Moreover, genetic associations of melanocortin system and melanogenesis genes with psoriasis were shown in a separate study where MC4R displayed the strongest statistical result [17].

Opioids play important roles in cutaneous nociception, immunomodulation and signal transduction. The system of endogenous opioids in the skin includes several neuromediators, which derive from four different precursors, namely, POMC, proenkephalin (PENK), prodynorphin (PDYN), and prepronociceptin (PNOC), and their receptors [18,19]. The importance of POMC in the context of the melanocortin system was already discussed above, but it can additionally be processed into β-endorphin. The many functions of β-endorphin include central and peripheral control of pain, sexual behavior and mechanisms of reward, while it has also been shown to inhibit peripheral pro-inflammatory mediators [20,21]. The data on PENK, PDYN and PNOC in relation to skin homeostasis is less abundant. Still, the cutaneous expression of their products and respective receptors has been detected, suggesting functional roles in tissue regeneration and skin ageing [18,22]. In relation to immune regulation in the skin, the opioid system has been implicated in various processes such as cytokine secretion, T-lymphocyte proliferation and dendritic cell maturation [18]. Opioids also serve as an important link between inflammatory mediators and pain sensation by counteracting the former and inducing analgesia [23]. With regard to inflammatory skin disorders, we have previously reported altered expression of PNOC and its receptor in vitiligo [24].

**Figure 1:**
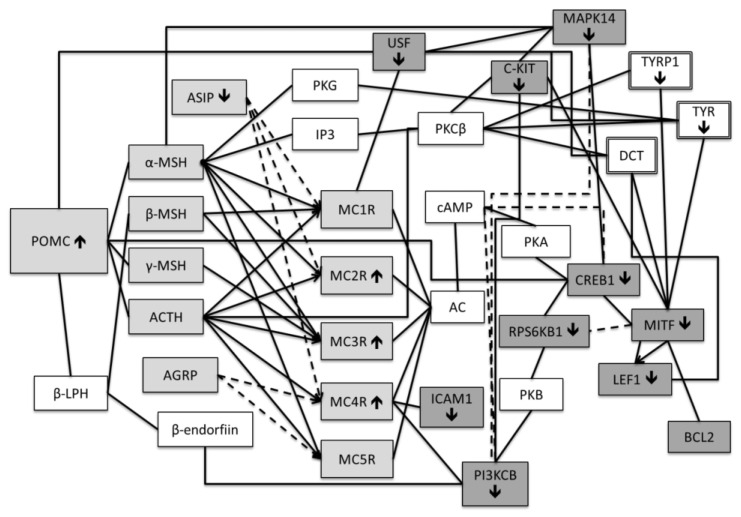
Main known regulatory links between the genes of the melanocortin system and enzymes of melanogenesis. ↑—up-regulation of mRNA in lesional psoriasis skin. ↓—down-regulation of mRNA in lesional psoriasis skin. In the case of POMC, MC2-4R, ASIP, TYR and TYRP1, the results are from our previous study [16]. The components of the melanocortin system are indicated with light gray and the associated factors that were investigated in this study are indicated with dark gray. Double borders indicate melanogenesis enzymes. 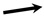 Receptor agonist 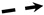 Receptor antagonist 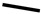 Positive regulation 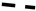 Negative regulation.

**Figure 2:**
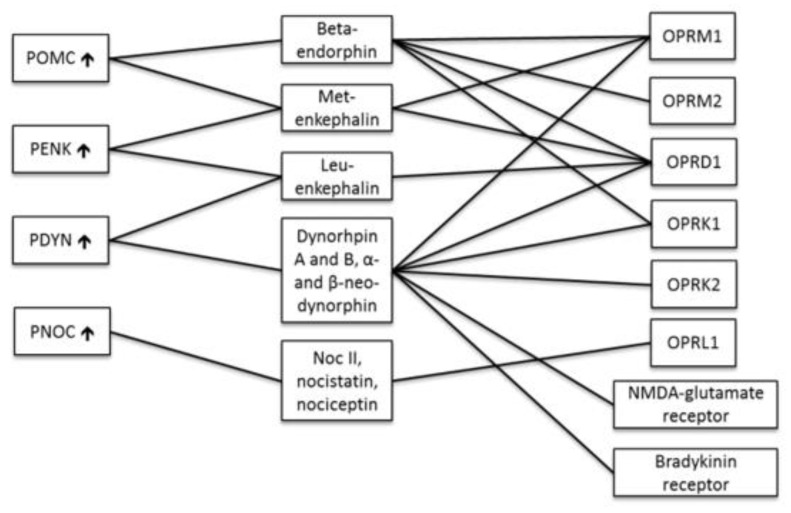
Endogenous opioids and receptors. Opioid precursor genes are on the left, opioid peptides in the middle and their receptors on the right. POMC, proopiomelanocortin; PENK, proenkephalin; PDYN, prodynorphin; PNOC, prepronociceptin; OPRM1 and -2, opioid receptor mu 1 and 2; OPRD1, opioid receptor delta 1; OPRK1 and -2, opioid receptor kappa 1 and 2; OPRL1, opioid receptor like-1; NMDA-glutamate receptor, N-methyl-D-aspartic acid-glutamate receptor. ↑—upregulation of mRNA in lesional psoriasis skin. In the case of POMC, the result is from our previous study [16]. The up-regulation of PENK was detected in lesional skin compared to non-lesional psoriasis skin.

Considering our former findings describing the altered expression of melanocortin genes and melanogenesis enzymes in psoriasis, aberrations could be suspected in the factors involved in signal transduction between them. Therefore, the first intention was to investigate the mRNA expression of the respective genes, including cAMP responsive element binding protein 1 (CREB1), microphthalmia-associated transcription factor (MITF), B-cell lymphoma 2 (BCL2), lymphoid enhancer-binding factor 1 (LEF1), upstream transcription factor 1 (USF1), mitogen-activated protein kinase 14 (MAPK14), intercellular adhesion molecule 1 (ICAM1), phosphatidylinositol-4,5-bisphosphate 3-kinase catalytic subunit beta (PIK3CB), ribosomal protein S6 kinase B1 (RPS6KB1), KIT proto-oncogene receptor tyrosine kinase (KIT), nuclear receptor related 1 protein (NURR1) and attractin (ATRN) (Figure 1). Due to the presence of endogenous opioids in the skin and their link to inflammation, the second aim was to evaluate the mRNA expression of PENK, PDYN, PNOC, opioid receptor kappa 1 (OPRK1), and opioid related nociceptin receptor 1 (OPRL1) (Figure 2).

## 2. Results

### 2.1. mRNA Expression in Lesional, Non-Lesional, and Control Skin

**Figure 3:**
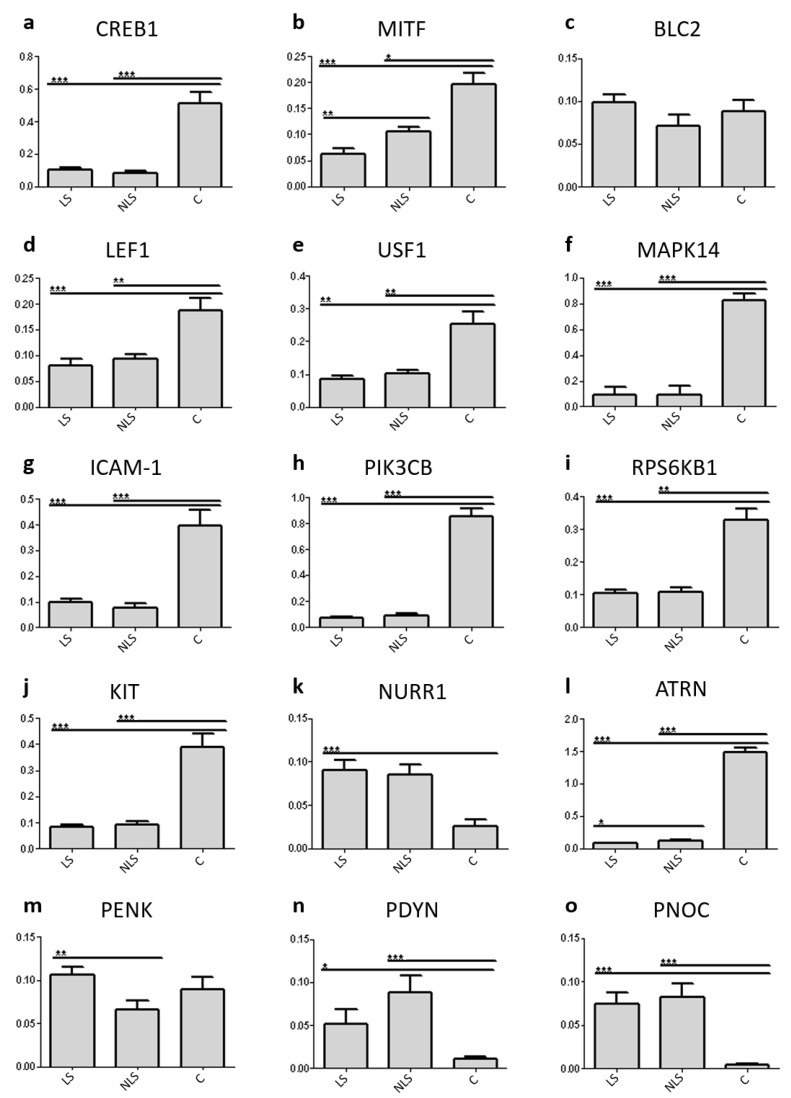
The mRNA expression levels of studied genes in skin biopsies comparing groups of psoriasis lesional skin (LS), psoriasis non-lesional skin (NLS) and skin of healthy control subjects (C). (**a**–**l**) Genes associated with intracellular signaling between melanocortin system and enzymes involved in melanin synthesis. (**m**–**o**) Endogenous opioid system genes. Results are displayed as mean ± SEM. * *p* < 0.05; ** *p* < 0.01; *** *p* < 0.001.

The mRNA expression levels of twelve genes related to intracellular signal transduction between the melanocortin system and enzymes of melanogenesis were measured together with five genes of endogenous opioid system (Figure 1, Figure 2 and Figure 3).

CREB1 mRNA expression in psoriasis lesional skin was 4.9-fold lower (*p* < 0.001; Figure 3a) and in non-lesional skin of psoriasis patients it was 6.3-fold lower (*p* < 0.001; Figure 3a) when compared to healthy control skin.

Statistically significant differences between all of the study groups were established for the mRNA expression of MITF gene. In psoriasis lesional skin, MITF mRNA expression was 3.2-fold lower (*p* < 0.001; Figure 3b) and in non-involved skin it was 1.9-fold lower (*p* < 0.05; Figure 3b) compared with the skin of healthy control subjects. In psoriasis lesional skin, a 1.7-fold decrease (*p* < 0.01; Figure 3b) in MITF mRNA expression was established when compared with psoriasis non-lesional skin.

No statistically significant differences in BCL2 mRNA expression were detected (Figure 3c).

LEF1 expression level was decreased in psoriasis lesional and non-lesional skin compared with healthy control skin. The LEF1 mRNA expression was 2.3-fold lower in lesional skin of patients with psoriasis (*p* < 0.001; Figure 3d) and 2.0-fold lower in psoriasis non-lesional skin (*p* < 0.01; Figure 3d) compared with healthy control subjects.

USF1 mRNA expression level in lesional skin of psoriasis patients was 3.0-fold lower (*p*< 0.01; Figure 3e) and in non-lesional skin it was 2.5-fold lower (*p* < 0.01; Figure 3e) than in the skin of healthy controls.

MAPK14 mRNA expression was 9.0-fold lower (*p* < 0.001; Figure 3f) in the lesional skin of psoriasis patients and 8.6-fold lower (*p* < 0.001; Figure 3f) in psoriasis non-lesional skin than in the skin of healthy control subjects.

ICAM1 mRNA expression level in psoriasis involved skin was 4.0-fold lower (*p* < 0.001; Figure 3g) and in non-involved skin it was 5.0-fold lower (*p* < 0.001; Figure 3g) compared with healthy control skin.

PIK3CB mRNA expression was 12.2-fold lower in psoriasis lesional skin when compared to healthy control skin (*p* < 0.001; Figure 3h) and 9.9-fold lower in non-lesional psoriasis skin compared with control skin samples (*p* < 0.001; Figure 3h).

Decreased RPS6KB1 mRNA expression in lesional as well as in non-lesional skin of psoriasis patients was established. In psoriasis lesional skin RPS6KB1 mRNA expression was 3.2-fold lower (*p* < 0.001; Figure 3i) and in psoriasis non-lesional skin it was 3.0-fold lower (*p* < 0.05; Figure 3i) compared with healthy control skin.

KIT mRNA expression level in lesional skin of psoriasis patients was 4.6-fold lower (*p* < 0.001; Figure 3j) and in psoriasis non-involved skin it was 4.2-fold lower (*p* < 0.001; Figure 3j) than in the skin of healthy control subjects.

In lesional skin of psoriasis patients a 3.6-fold increase (*p* < 0.001; Figure 3k) in NURR1 mRNA expression was detected when compared with healthy control samples.

ATRN mRNA expression in lesional skin of psoriasis patients was 19.5-fold lower (*p* < 0.001; Figure 3l) and in psoriasis non-lesional skin it was 12.4-fold lower (*p* < 0.001; Figure 3l) compared with healthy control skin. ATRN mRNA expression level was 1.6-fold higher in non-lesional skin of psoriasis patients when compared with lesional skin of psoriasis patients (*p* < 0.05; Figure 3l).

In psoriasis lesional skin, PENK mRNA expression was 1.6 times higher (*p* < 0.01; Figure 3m) when compared to psoriasis non-lesional skin. There were no statistically significant changes between other study groups.

In psoriasis-affected skin, a 4.7-fold increase (*p* < 0.05; Figure 3n) and in psoriasis non-involved skin an 8.0-fold increase (*p* < 0.001; Figure 3n) in the expression of PDYN mRNA was detected when compared with healthy control skin.

Increased PNOC mRNA expression in lesional and non-lesional psoriasis skin compared with healthy controls was established. PNOC mRNA expression was 16.4-fold higher (*p* < 0.001; Figure 3o) in lesional skin and 18.0-fold higher (*p* < 0.001; Figure 3o) in non-lesional skin compared with the skin of healthy control subjects.

OPRK1 and OPRL1 mRNA expression did not reach the detection threshold when 250–500 ng of cDNA was applied to the QRT-PCR reaction.

### 2.2. Expression Level Interactions

Spearman rank correlation analysis was used to study possible interactions between the expression levels of studied genes (Table 1, Table 2 and Table 3). In psoriasis involved skin (Table 1), statistically significant positive correlations were found between the expression levels of PNOC and USF1 (r = 0.58, *p* < 0.05), PNOC and ICAM1 (r = 0.59, *p* < 0.05) and USF1 and ICAM1 (r = 0.51, *p* < 0.05). Statistically significant negative correlations were established between MITF and RPS6KB1 (r = −0.53, *p* < 0.05), CREB1 and PENK (r = −0.48, *p* < 0.05), and MAPK14 and PENK (r = −0.48, *p* < 0.05).

In psoriasis uninvolved skin (Table 2), positive correlations were found between the expression levels of CREB1 and LEF1 (r = 0.66, *p* < 0.05), USF1 and PNOC (r = 0.81, *p* < 0.05), MITF and ATRN (r = 0.58, *p* < 0.05), MITF and NURR1 (r = 0.56, *p* < 0.05) and MITF and MAPK14 (r = 0.75, *p* < 0.01). Negative correlations were found between CREB1 and PDYN (r = −0.89, *p* < 0.01), PIK3CB and PNOC (r = −0.81, *p* < 0.05), PIK3CB and MITF (r = −0.64, *p* < 0.05), RPS6KB1 and KIT (r = −0.71, *p* < 0.05) and ICAM1 and PDYN (r = −0.76, *p* < 0.05).

Additionally, in healthy control skin (Table 3), positive correlations were found between the expression levels of MITF and PIK3CB (r = 0.53, *p* < 0.01). Negative correlations were found between PDYN and PENK (r = −0.73, *p* < 0.05) and RPS6KB1 and MAPK14 (r = −0.51, *p* < 0.05).

## 3. Discussion

As the results indicate, the genes associated with the intracellular signaling system linking melanocortin receptors with enzymes involved in melanin synthesis were generally suppressed in psoriasis. At the same time, the expression levels of studied endogenous opioid system genes were increased in psoriatic skin.

Cyclic adenosine monophosphate/protein kinase A (cAMP/PKA) is the main pathway in human melanocytes through which signal from the melanocortin system reaches the melanogenesis enzymes TYR, TYRP1 and dopachrome tautomerase (DCT). It is additionally modulated by the Wnt and MAPK pathways [25]. Specifically, cAMP activates PKA, and PKA phosphorylates and activates CREB1 transcription factor, leading to the up-regulation of MITF expression. MITF then up-regulates TYR, TYRP1 and DCT [26]. LEF1 is a transcription factor that participates in the Wnt signaling pathway and also stimulates the transcription of tyrosinase-related genes through its effects on MITF [27]. USF1 regulates pigmentation in melanocytes by binding to the promoter of TYR and activating its transcription when phosphorylated by p38 MAPK (includes p38α/MAPK14, p38β/MAPK11, p38γ/MAPK12, and p38δ/MAPK13), which itself has been stimulated by ultraviolet irradiation [28]. It is also known that ICAM1 cross-linking triggers activation of p38 MAPK [29]. Another outcome of signaling via p38 MAPK is the activation of CREB1, thus promoting MITF expression, and consequently, enhanced TYR expression [28]. Herein, we found decreased levels of CREB1, MITF, LEF1, USF1, ICAM1, and MAPK14 mRNA expression in lesional and non-lesional psoriatic skin compared to healthy control skin. This may result from elevated proinflammatory cytokines in psoriasis-TNF, IL-17, IL-1 and IL-6, which are known to inhibit melanogenesis and specifically PKA, MITF, TYR and DCT [12,14,30]. Likewise, another inflammatory cytokine associated with psoriasis, interferon gamma, has been shown to suppress CREB, MITF, p38 MAPK and melanogenesis [31,32,33]. These current findings are in agreement with our previous study where we found lowered expression of TYR and TYRP1 mRNA in psoriatic skin compared to healthy controls [16]. It should be noted however, that while we reported similarly lowered MAPK14 levels in the case of inflammatory skin disease, vitiligo, its expression in psoriasis has been found to be elevated according to preceding studies by other authors [34,35,36].

PI3K lipid kinases have been implicated in many physiological processes, such as proliferation, survival, intracellular traffic and cell differentiation [37]. We established that PIK3CB mRNA expression was significantly lower in psoriatic skin. As PI3K/Akt pathway activation leads to a decrease in cell death [38], lowered PIK3CB level in psoriatic skin probably causes increased susceptibility of skin cells to apoptosis. In our opinion, PI3K/Akt down-regulation in psoriasis should be viewed as a compensatory protective mechanism, which is activated by cells to enhance cell death, especially among the abundantly produced keratinocytes. In relation to melanogenesis, the inhibition of PI3K/Akt has been shown to increase melanin content but there have also been mixed results in this regard [39].

RPS6KB1 encodes 70 kDa ribosomal S6 kinase (p70S6K) that acts downstream of PI3K. Numerous mitogens, growth factors and hormones activate the p70S6K. Its functions include regulating cell motility, a cellular response that is important in tumor metastases, the immune response and tissue repair [40]. It has been shown that cAMP triggers a significant inhibition of PI3K activity and a strong blockage of p70S6K activity [41]. Since selective inhibitors of PI3K and p70S6K increased melanin production and TYR activity, it was proposed that the melanogenic effect of cAMP is at least partially mediated by its suppression of PI3K/p70S6K pathway. We found lower expression of RPS6KB1 mRNA, similar to the expression of PIK3CB mRNA, in lesional as well as non-lesional psoriatic skin compared to healthy controls, which is in contrast with our other findings that point to the reduction in melanogenesis.

KIT encodes a receptor tyrosine kinase protein named mast/stem cell growth factor receptor (SCFR), which stimulates both the MAPK and PI3K-Akt pathways; thus, in melanoma, mutations in KIT lead to deregulated growth and survival of cells [42]. It is known that in the melanocytes of vitiligo involved skin, the expression level of KIT is decreased and can be associated with the dysfunction and/or loss of these cells [43]. However, despite the ability of psoriasis-associated TNF and IL-17 to reduce KIT expression in melanocytes, the number of melanocytes in psoriasis lesional skin is increased [13,14]. Another function of KIT is the activation of the MAPK signaling pathway, which induces MITF and subsequent melanogenic enzymes [44]. Therefore, the presently found significant reduction in KIT mRNA in lesional and non-lesional psoriatic skin may primarily reflect the suppression of melanogenesis without concurrent loss of melanocytes.

NURR1, as well as other nuclear receptor subfamily 4 (NR4A) members play an important role in central hypothalamic–pituitary–adrenal axis regulation including transcriptional effects on CRH, POMC, and enzymes that control cortisol production. It has been suggested that in human melanoma cells, CRH and urocortin regulate TYRP1 gene expression via NURR1/NUR77 production [45]. NURR1 also contributes to the regulation of inflammation through its effects on peripheral CRH signaling in human inflammatory joint diseases such as psoriatic arthropathia [46]. It has been shown previously that NURR1 mRNA expression is up-regulated in psoriatic involved skin compared to uninvolved skin and healthy control skin, while treatment with TNF-α inhibitors reduces NURR1 levels, suggesting that the clinical benefits of TNF-α inhibition may be mediated through altered NURR1 activity [47]. Our findings are partly in agreement with that; we established a statistically significant increase in NURR1 mRNA expression in psoriasis involved skin only when compared to healthy control skin.

ATRN1 is a type one transmembrane protein that behaves as an accessory receptor for melanocortin receptor antagonist ASIP [48]. ATRN1 participates in melanin synthesis in melanocytes since ASIP-MC1R signaling includes a cAMP-independent pathway that passes through ATRN [49]. Our results show decreased expression of ATRN mRNA in lesional skin compared to non-lesional and healthy skin. Also, in non-lesional skin the expression of ATRN mRNA was significantly lower than in control skin. In our previous study, we found decreased expression of ASIP mRNA in psoriatic lesional and non-lesional skin [16]. Consequently, the decrease in ATRN mRNA could be involved in the hypopigmentation that occurs at the healing of psoriatic lesions.

Our finding of PENK upregulation in lesional psoriatic skin is in agreement with an earlier study showing an increase in PENK mRNA and the increased expression of PENK peptides met- and leu-enkephalin in psoriatic skin [50]. The activation of PENK was also achieved by subjecting keratinocytes to inflammatory stimuli and is considered as a part of the epidermal innate immune response, which is itself known to have a role in psoriasis etiology [50,51]. Our finding of the increased expression of PDYN contributes to the findings of Taneda et al. [52], who in 2011 demonstrated a decrease in dynorphin A and κ-opioid receptor levels in the epidermis of pruritic psoriasis patients, as this overexpression may be an attempt to compensate for the decrease in dynorphin A. Unfortunately, our attempt to analyze the expression of κ-opioid receptor gene OPRK1 was not successful as the mRNA level did not reach the detection threshold. Previously, our research group has reported an increase in the mRNA expression of PNOC and its receptor ORPL1 in vitiligo involved skin compared to healthy controls [24]. In the current study, PNOC mRNA was significantly up-regulated in the lesional as well as non-lesional skin of psoriasis patients compared to healthy controls, but the PNOC receptor gene OPRL1 did not reach the detection threshold. It has been demonstrated that the PNOC product nociceptin may induce the production of inflammatory mediators and cytokines can, in turn, suppress its precursor prepro-nociceptin [53,54]. There is currently no consensus on the role of nociceptin in inflammatory and immune responses, which could depend on the tissue and condition studied, but its ability to affect chemotaxis, cellular and humoral immunity and the expression of proinflammatory cytokines may exacerbate the inflammatory environment of psoriasis [54].

In conclusion, the genes related to signal transduction between the melanocortin system and melanogenesis enzymes were generally down-regulated, indicating the reduction in melanin synthesis in psoriasis and explaining the hypopigmentation that is observed after the healing of psoriatic patches. On the other hand, the increase in endogenous opioids implies their involvement in inflammatory regulation and activated nociceptive function in psoriasis. When considering the former set of findings, it should be noted that the measured genes are involved in different processes in addition to melanin production and whole skin punch biopsies were used that include other cells besides melanocytes. Since it has been demonstrated how individual cell types can display distinct expressional patterns in psoriasis [55,56], it is possible that other cells and mechanisms contribute to the changes observed here. Still, on the grounds of reduced melanogenesis and the association of these genes to melanin production, an interpretation that links them together can be suggested as an overarching explanation.

## 4. Materials and Methods

The Research Ethics Committee of the University of Tartu approved the protocols and informed consent forms of this study. Written informed consent was signed by all of the participants.

### 4.1. Patients and Healthy Controls

The patients as well as the control subjects in the study were Caucasians living in Estonia. Forty-four (8 females; 36 males) unrelated patients with plaque psoriasis were recruited at the Clinic of Dermatology, Tartu University Hospital. Their mean age was 43.3 years (range 18–64 years) and the mean age of psoriasis onset was 26.96 years. The mean PASI score was 21.8 (range 2–69). Thirty-seven patients had a PASI score over 10 (moderate and severe disease) and seven patients had a PASI score less than 10 (mild disease) [57]. Twenty-nine of the patients had psoriatic nail involvement, ten had psoriatic arthropathy, and sixteen patients had a positive family history of psoriasis. The patient groups did not receive any local or systemic treatment for at least a month prior to taking the biopsies. Healthy volunteers were recruited as a control group, which included 75 individuals (23 male, 52 female) with an average age of 35.4 years (range 21–70) and all were free from positive family history of psoriasis and other dermatoses.

### 4.2. Skin Samples and Quantitative Real-Time-PCR

Two four-millimeter punch biopsies from both lesional (LS) and non-lesional (NLS) skin were obtained from the patients. One skin biopsy (4 Ø mm) was obtained from healthy control subjects. All of the biopsies were taken from non-sun-exposed skin. All probands had skin phototype II or III according to Fitzpatrick classification. Biopsies were instantly snap-frozen and stored at −80 °C until RNA extraction.

RNeasy Fibrous Tissue Mini Kit (QIAGEN Sciences, Germantown, MA, USA) was used according to the manufacturer’s protocol for total RNA extraction from skin biopsies. Biopsies were homogenized by T 10 basic homogenizer (IKA Labortechnik, Staufen im Breisgau, Germany). Extracted RNA was dissolved in RNase free water and stored at −80 °C until cDNA synthesis. RNA quality was controlled using the NanoDropTM 1000 (Thermo Fisher Scientific, Waltham, MA, USA). According to the manufacturer’s protocol, 250–500 ng of total RNA, oligoT18 primer and Superscript III reverse transcriptase (Invitrogen Corp., Carlsbad, CA, USA) were used for cDNA synthesis.

cDNA was used as a template for TaqMan^®^ QRT-PCR analysis in the ABI Prism 7900HT Sequence Detection System (Applied Biosystems, Foster City, CA, USA). Reactions were carried out in 10 µL reaction volumes in four replicates. Two primers and a labelled probe were used to detect the mRNA expression level of the reference gene HPRT-1 (hypoxanthine phosphoribosyl-transferase-1) (HPRT-1 exon 6, 5′-GACTTTGCTTTCCTTGGTCAGG-3′; HPRT-1 exon 7, 5′-AGTCTGGCTTATATCCAACACTTCG-3′; VIC-5′-TTTCACCAAGCTTGCGACCTTGA-3′-TAMRA). The expression levels of CREB1, MITF, BCL2, LEF1, USF1, p38α/MAPK14, ICAM1, PIK3CB, RPS6KB1, KIT, NURR1, ATRN, PENK, PDYN, PNOC, OPRK1 and OPRL1 were detected by applying TaqMan-QRT-PCR method using TaqManTM Gene Expression Assays (Applied Biosystems, Foster City, CA, USA). The assay mixes used were Hs00231713_m1 (CREB1), Hs00165156_m1 (MITF), Hs0060823_m1 (BCL2), Hs00212390_m1 (LEF1), Hs00273038_m1 (USF1), Hs00176247_m1 (p38α/MAPK14), Hs00164932_m1 (ICAM1), Hs00178872_m1 (PIK3CB), Hs00177357_m1 (RPS6KB1), Hs00174029_m1 (KIT), Hs01118813_m1 (NURR1), Hs00390610_m1 (ATRN), Hs00175049_m1 (PENK), Hs00225770_m1 (PDYN), Hs00173823_m1 (PNOC), Hs00175127_m1 (OPRK1) and Hs00173471_m1 (OPRL1).

### 4.3. Statistical Analyses

A comparative Ct method (Δ^Ct^ value) was used for mRNA quantification, where the amount of target transcript was normalized according to the level of endogenous reference HPRT-1. The data of studied genes that followed a normal distribution were parametrically tested by unpaired t-tests and the data that did not follow a normal distribution were tested by the Mann–Whitney U test using Graphpad Prism 4 software (GraphPad Software, San Diego, CA, USA). For all tests, a *p* value < 0.05 was considered significant.

## Figures and Tables

**Table 1:** Results of correlation analysis of gene expression in lesional skin biopsies of psoriasis patients. Spearman rank correlation coefficient (Spearman r) was used; * *p* < 0.05.

	BLC2	CREB1	LEF1	PNOC	USF1	PIK3CB	RPS6KB1	MITF	MAPK14	NURR1	ATRN	KIT	ICAM1	PDYN	PENK
BLC2	1.00														
CREB1	−0.16	1.00													
LEF1	−0.13	−0.03	1.00												
PNOC	−0.26	−0.22	0.22	1.00											
USF1	−0.11	−0.17	−0.20	0.58 *	1.00										
PIK3CB	−0.21	−0.14	0.05	−0.08	0.33	1.00									
RPS6KB1	0.00	0.27	−0.03	0.06	0.11	−0.11	1.00								
MITF	0.47	−0.13	−0.08	−0.38	−0.24	−0.31	−0.52 *	1.00							
MAPK14	−0.11	0.46	0.31	−0.22	−0.16	−0.16	0.26	0.04	1.00						
NURR1	0.11	−0.02	−0.01	0.21	0.05	−0.14	0.16	0.01	0.06	1.00					
ATRN	−0.02	−0.09	−0.32	0.34	0.02	−0.42	0.10	0.03	−0.22	−0.24	1.00				
KIT	−0.10	0.22	−0.09	−0.07	−0.06	−0.15	−0.17	0.09	−0.28	0.44	−0.27	1.00			
ICAM1	0.02	−0.20	−0.20	0.59 *	0.51 *	−0.06	0.33	−0.21	0.03	0.41	0.05	−0.09	1.00		
PDYN	0.25	0.24	−0.23	0.14	0.10	−0.34	−0.10	0.12	0.03	0.08	−0.15	0.06	0.18	1.00	
PENK	0.12	−0.47 *	−0.42	0.22	0.23	0.16	−0.29	0.12	−0.48 *	0.30	−0.15	0.11	0.27	0.46	1.00

**Table 2:** Results of correlation analysis of gene expression in non-lesional skin biopsies of psoriasis patients. Spearman rank correlation coefficient (Spearman r) was used; * *p* < 0.05; ** *p* < 0.01.

	BLC2	CREB1	LEF1	PNOC	USF1	PIK3CB	RPS6KB1	MITF	MAPK14	NURR1	ATRN	KIT	ICAM1	PDYN	PENK
BLC2	1.00														
CREB1	−0.40	1.00													
LEF1	−0.38	0.65 *	1.00												
PNOC	0.08	−0.38	−0.53	1.00											
USF1	−0.36	−0.13	−0.11	0.81 *	1.00										
PIK3CB	0.05	0.36	0.15	−0.81 *	−0.02	1.00									
RPS6KB1	0.05	0.34	−0.34	0.11	0.23	0.18	1.00								
MITF	0.34	−0.42	−0.10	0.40	−0.31	−0.64 *	0.04	1.00							
MAPK14	0.26	−0.11	0.17	0.45	−0.26	−0.51	−0.23	0.75 **	1.00						
NURR1	0.36	−0.09	−0.01	0.05	0.17	−0.04	0.58	0.55 *	0.23	1.00					
ATRN	0.11	−0.40	−0.28	0.40	0.09	−0.31	0.16	0.58 *	0.36	0.49	1.00				
KIT	−0.23	0.06	0.05	−0.21	−0.17	0.15	−0.71 *	−0.27	0.19	−0.51	−0.35	1.00			
ICAM1	0.10	0.29	0.27	−0.42	−0.23	0.15	−0.24	−0.37	−0.22	0.09	−0.33	0.34	1.00		
PDYN	−0.39	−0.89 **	−0.51	0.39	0.57	−0.18	0.02	0.53	0.31	0.02	0.19	−0.13	−0.76 *	1.00	
PENK	0.46	−0.07	−0.33	0.04	−0.22	−0.06	0.22	0.05	−0.03	0.18	0.08	−0.10	0.43	−0.25	1.00

**Table 3:** Results of correlation analysis of gene expression in skin biopsies of healthy controls. Spearman rank correlation coefficient (Spearman r) was used. * *p* < 0.05; ** *p* < 0.01.

	BLC2	CREB1	LEF1	PNOC	USF1	PIK3CB	RPS6KB1	MITF	MAPK14	NURR1	ATRN	KIT	ICAM1	PDYN	PENK
BLC2	1.00														
CREB1	0.16	1.00													
LEF1	−0.32	0.44	1.00												
PNOC	−0.28	0.62	0.05	1.00											
USF1	0.42	0.12	0.22	0.02	1.00										
PIK3CB	0.40	0.29	0.36	0.08	0.38	1.00									
RPS6KB1	−0.08	0.15	−0.21	−0.33	−0.05	−0.34	1.00								
MITF	0.23	−0.18	−0.15	−0.43	0.22	0.53 **	−0.12	1.00							
MAPK14	0.29	0.19	0.37	0.28	0.40	0.36	−0.51 *	0.36	1.00						
NURR1	0.07	0.38	0.13	−0.05	−0.14	−0.08	−0.23	−0.25	−0.14	1.00					
ATRN	0.43	−0.48	−0.10	−0.09	0.14	0.50	−0.16	0.01	0.54	−0.46	1.00				
KIT	0.26	−0.40	0.10	0.14	−0.27	0.27	−0.56	−0.25	0.51	−0.01	0.57	1.00			
ICAM1	0.13	−0.28	0.02	−0.32	−0.04	−0.26	0.10	−0.22	−0.30	−0.10	−0.36	−0.03	1.00		
PDYN	−0.15	0.28	−0.22	0.30	0.00	−0.14	−0.21	−0.31	−0.09	0.11	0.08	−0.09	0.09	1.00	
PENK	0.13	−0.35	0.23	−0.28	0.14	0.33	−0.09	0.34	0.40	−0.41	0.06	0.37	0.22	−0.73 *	1.00

## Data Availability

The datasets used and/or analyzed for the study are available from the corresponding author on reasonable request.
